# Histidine-Bound Dinitrosyl Iron Complexes: Antioxidant and Antiradical Properties

**DOI:** 10.3390/ijms242417236

**Published:** 2023-12-07

**Authors:** Konstantin B. Shumaev, Olga V. Kosmachevskaya, Elvira I. Nasybullina, Enno K. Ruuge, Elena I. Kalenikova, Alexey F. Topunov

**Affiliations:** 1 Bach Institute of Biochemistry, Research Center of Biotechnology, Russian Academy of Sciences, 119071 Moscow, Russia; tomorov@mail.ru (K.B.S.); rizobium@yandex.ru (O.V.K.); lvirus198709@rambler.ru (E.I.N.); 2 E.I. Chazov National Medical Research Center of Cardiology, 121552 Moscow, Russia; ruuge@mail.ru; 3 Faculty of Fundamental Medicine, Lomonosov Moscow State University, 119991 Moscow, Russia; ekaleni@fbm.msu.ru

**Keywords:** nitric oxide, dinitrosyl iron complexes, carnosine, albumin, histidine, prooxidant, antioxidant, peroxidation

## Abstract

Dinitrosyl iron complexes (DNICs) are important physiological derivatives of nitric oxide. These complexes have a wide range of biological activities, with antioxidant and antiradical ones being of particular interest and importance. We studied the interaction between DNICs associated with the dipeptide L-carnosine or serum albumin and prooxidants under conditions mimicking oxidative stress. The ligands of these DNICs were histidine residues of carnosine or His39 and Cys34 in bovine serum albumin. Carnosine-bound DNICs reduced the level of piperazine free radicals in the reaction system containing *tert*-butyl hydroperoxide (*t*-BOOH), bivalent iron ions, a nitroxyl anion donor (Angeli’s salt), and HEPES buffer. The ability of carnosine DNICs to intercept organic free radicals produced from *t*-BOOH decay could lead to this effect. In addition, carnosine DNICs reacted with the superoxide anion radical (O_2_^•−^) formed in the xanthine/xanthine oxidase enzymatic system. They also reduced the oxoferryl form of the heme group formed in the reaction of myoglobin with *t*-BOOH. DNICs associated with serum albumin were found to be rapidly destroyed in a model system containing metmyoglobin and t-BOOH. At the same time, these protein DNICs inhibited the *t*-BOOH-induced oxidative degradation of coenzymes Q_9_ and Q_10_ in rat myocardial homogenate. The possible mechanisms of the antioxidant and antiradical action of the DNICs studied and their role in the metabolism of reactive oxygen and nitrogen species are discussed.

## 1. Introduction

Physiological derivatives of nitric oxide (NO^•^) including dinitrosyl iron complexes (DNICs) perform numerous functions in living systems [1,2,3,4]. DNICs have a wide range of biological activities: they participate in NO^•^ scavenging, have hypotensive, cytoprotective, and antioxidant properties, inhibit platelet aggregation, increase the elasticity of erythrocyte membranes, and promote wound healing [3,5,6,7,8,9,10]. Most of these activities are related to the ability of DNICs to act as NO^•^ donors.

DNICs, whose ligands, along with NO^•^, are low-molecular-weight or protein-bound thiols, are the most studied [3,11,12,13]. Some thiol-containing DNICs release nitrosonium (NO^+^) cations, responsible for their cytotoxic and antiviral (including SARS-CoV-2) action [14].

**Figure 1:**
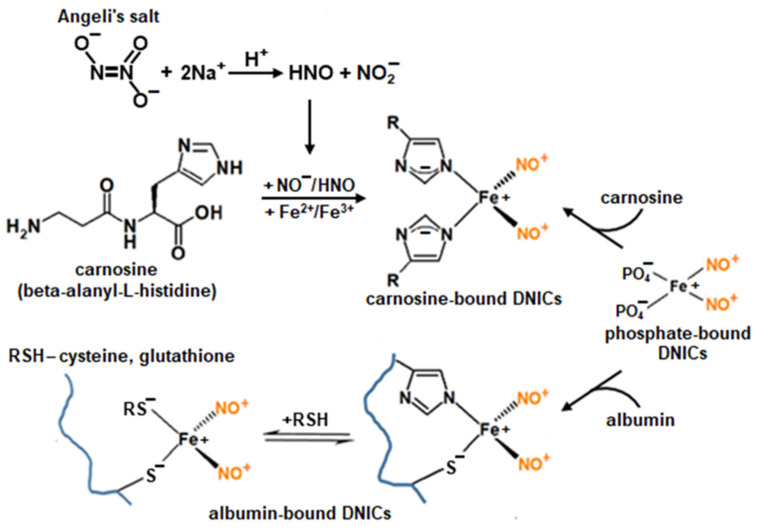
Formation of dinitrosyl iron complexes (DNICs) with histidine ligands of carnosine and albumin. The role of phosphate DNICs and Angeli’s salt (sodium trioxidinitrate).

The iron in DNICs can also be coordinated with phosphate anions, with the nitrogen of the histidine imidazole ring, and with some other ligands [3,11,15,16]. DNICs, whose ligands are histidine residues in peptides and proteins (Figure 1), have received the most attention. Particularly, the most studied are DNICs associated with carnosine or serum albumin as they are defined by diverse functions and occur in large amounts in living organisms. 

The dipeptide carnosine (β-alanyl-histidine) chelates transition metal ions, regulates calcium metabolism, has anti-inflammatory and immunostimulating activity, and functions as an intracellular buffer, neurotransmitter, and geroprotector [17,18,19,20,21]. Moreover, this dipeptide effectively protects proteins against non-enzymatic glycation, acting as an anti-glycating agent [18,19,22,23]. 

Special attention has been paid to the antioxidant properties of carnosine [18,19,20,22,24,25]. They were detected in various systems modeling oxidative stress, including nerve tissue and cardiac muscle [17,19,20,21,23,26,27]. Thanks to these properties, carnosine can protect cells from oxidative damage and slow down their aging [28]. Dietary supplements on the basis of carnosine are produced to improve efficiency under physical activity, to slow down aging, as well as for immunomodulation and the treatment/prevention of diabetes and neurological disorders [28,29,30,31]. 

Research is underway to create compounds based on carnosine with therapeutic potential that combine the functions of the peptide and a conjugating fragment. These may be carnosine derivatives containing NO-donor substructures, e.g., NO-donor carnosine amides [32]. This class of substances is interesting as a potential remedy for the treatment of many chronic vascular and neurodegenerative diseases when the bioavailability of NO is decreased. They demonstrated antioxidant activity in vitro and protection against ischemia/reperfusion in vivo, as well as a vasodilation effect on rat aortic fragments [32]. 

The class of NO-donor carnosine derivatives also includes DNICs associated with carnosine. We have previously shown that carnosine in the presence of the nitroxyl anion (NO^−^) and iron ions participates in DNIC formation [16]. In that work, Angeli’s salt (sodium trioxidinitrate) was the donor of HNO (the protonated form of the nitroxyl anion) (Figure 1). However, the interaction of carnosine DNICs with reactive oxygen species (ROS) has not yet been studied in contrast with that of thiol-containing DNICs. Importantly, in skeletal muscles and nervous tissue, carnosine is present at high (millimolar) concentrations [19,21].

Mammalian serum albumin is the major protein in blood plasma, as well as a vital component of lymph, interstitial, and cerebrospinal fluids [33,34]. This protein is responsible for colloidal-osmotic (oncotic) plasma pressure, as well as for the binding and transport of low-molecular-weight compounds: fatty acids, carotenoids, heme, and metal ions [34,35,36]. Albumin is ROS-modified under oxidative stress, e.g., under ischemia/reperfusion conditions [34,36,37,38,39], and ischemically modified albumin contains the damaged metal-binding centers. In addition, the cysteine residue (Cys34) of serum albumin can be nitrosylated to form S-nitrosothiol [15,36,40,41]. Albumin-stabilized tetranitrosyl complexes with thiosulfate ligands produce an antioxidant effect [42]; however, these complexes are not physiological. 

Two types of DNICs can be associated with human and bovine serum albumins. In the first type, iron is coordinated with a Cys34 residue and a low-molecular-weight thiol, e.g., glutathione [15,35,41]. In the second type, the non-protein thiol ligand is replaced with the histidine residue of the albumin protein chain [6,41]. This complex associated with bovine serum albumin (BSA) has the formula (NO)_2_Fe(Cys34)(His39) [41]. Under pro-oxidant action, a low-molecular-weight thiol ligand of albumin DNICs is substituted with the His39 residue [6,7].

Since DNICs’ physiological effects, including their antioxidant action, largely depend on the reactivity of their constituent ligands, we aimed to study the effect of free radicals and other oxidants on DNICs associated with histidine residues of carnosine and albumin.

## 2. Results 

### 2.1. Interaction of Carnosine-Bound DNICs with Free Radicals and Other Oxidants in Model Systems

**Figure 2:**
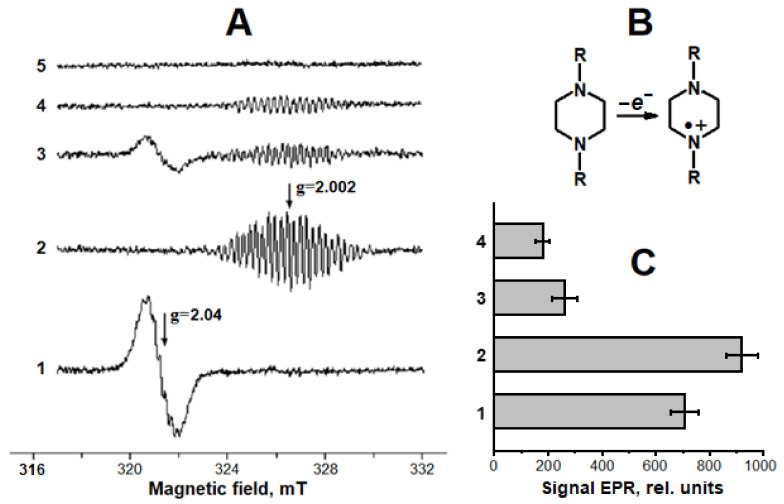
Interaction of carnosine DNICs with *tert*-butyl hydroperoxide (*t*-BOOH) and its effect on the formation of piperazine-derived cation radical. (**A**)—electron paramagnetic resonance (EPR) spectra of DNICs recorded after 12.5 min of incubating the reaction mixture containing 5 mM of Angeli’s salt, 1 mM of Fe^3+^, and 25 mM of carnosine (1); the same as (1), but without carnosine and 3 min after adding 4 mM of *t*-BOOH (2); the same as (1), 3 min after adding 4 mM of *t*-BOOH (3); 15.5 min incubation of the mixture containing only Angeli’s salt (4); and 3 min after mixing 1 mM of Fe^3+^ and 4 mM of *t*-BOOH (5). In all the cases, the reaction mixture contained 0.1 M of HEPES (pH 7.5). (**B**)—structural formula of piperazine cation radical. (**C**)—the intensity of EPR signals of piperazine cation radical: 1—reaction mixture contained Fe^2+^ ions and 4 mM of *t*–BOOH, and the spectra were recorded 3 min after mixing the components; columns 2–4 correspond to spectra 2–4 in panel (**A**).

In model systems containing carnosine, iron ions, and the donor of the nitroxyl anion, Angeli’s salt, DNICs were shown to be formed with iron bound to the imidazolate anion of the carnosine histidine residue [16]. Carnosine DNICs had a characteristic electron paramagnetic resonance (EPR) spectrum with a g-factor of 2.034 (Figure 2, spectrum 1). These DNICs were also formed when carnosine was added to DNICs with phosphate ligands. In this case, weakly bound phosphate anions in the complexes were replaced with carnosine.

The piperazine cation radical (Figure 2B) is known to be formed via single-electron HEPES oxidation by peroxynitrite [43]. In our experiments, the most intense formation of the piperazine cation radical occurred during the incubation of ferric ions (Fe^3+^) and the NO^−^ donor, Angeli’s salt, in HEPES buffer, followed by adding *t*-BOOH (Figure 2A, spectrum 2). In the reaction mixture containing Fe^2+^ ions, *t*-BOOH, and HEPES buffer, a slightly lower level of the free radical piperazine derivative was recorded (Figure 2C, column 1). The intensity of the EPR signal of this free radical was estimated by the amplitude of the central line of the EPR spectrum with a g-factor of 2.02. In the mixture containing only HEPES and Angeli’s salt, the piperazine cation radical was produced together with ONOO^−^ and with the reduction of Fe^3+^ ions to Fe^2+^ by the nitroxyl anion. In fact, in the presence of Fe^3+^, but without the NO^−^ donor, the piperazine cation radical was not formed (Figure 2A, spectrum 5).

HEPES can be oxidized by ONOO^−^ or *t*-BO^•^, and the input of the latter is greater. Thus, in the applied model of free radical oxidation, the nitroxyl anion acts as a pro-oxidant. 

**Figure 3:**
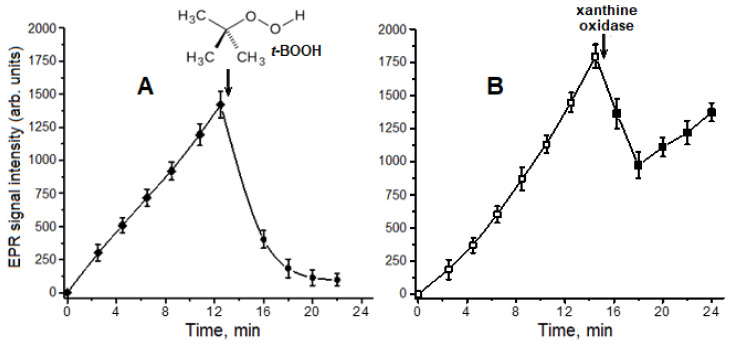
Kinetics of carnosine DNIC formation, and the effects of *t*-BOOH addition to the reaction mixture (**A**) or enzymatic generation of superoxide radical (**B**) on DNIC level. The reaction mixture contained 0.1 M of HEPES buffer, 5 mM of Angeli’s salt, 1 mM of Fe^3+^, and 25 mM of carnosine. (**A**)—after 12.5 min incubation, 4 mM of *t*-BOOH was added to the mixture, and the structural formula of *t*-BOOH is also shown; (**B**)—reaction mixture also contained 1.5 mM of xanthine. After 14.5 min of incubation, xanthine oxidase (0.6 U/mL) was added to the mixture.

We studied the kinetics of carnosine-bound DNIC formation when carnosine was incubated with the NO^−^ donor and ferric ions Fe^3+^ (Figure 3A). Adding *t*-BOOH to the reaction mixture noticeably decreased the DNICs’ EPR signal (Figure 2A, spectra 2 and 3; Figure 3A). At the same time, the production of the piperazine cation radical was inhibited (Figure 2, spectra 2 and 5). We assume that the destruction and antiradical effect of carnosine DNICs result from their interaction with pro-oxidants formed in the used model system. Furthermore, the antioxidant effect of DNICs may stem from the iron ions of these complexes catalyzing free radical peroxidation processes [44,45].

The superoxide anion radical (O_2_^•−^) is a precursor of other ROS and reactive nitrogen species, e.g., peroxynitrite. We have previously shown the effect of O_2_^•−^ on thiol-containing DNICs [9]. O_2_^•−^ production is also known to increase under conditions simulating carbonyl stress and diabetic hyperglycemia [46,47]. Herein, we studied the carnosine-bound DNICs’ interaction with O_2_^•−^, enzymatically generated in the xanthine–xanthine oxidase system. Xanthine oxidase (xanthine oxidoreductase, EC 1.17.3.2) catalyzes the formation of O_2_^•−^ in the single-electron oxidation reaction of xanthine with molecular oxygen. The reaction mixture also contained Angeli’s salt and Fe^3+^ ions. After xanthine oxidase was added, the increase in the carnosine-bound DNIC content was replaced with a drop in their level, but then it began to increase again, although at a slower rate (Figure 3B). These data show that in contrast with *t*-BOOH action (Figure 3A), when O_2_^•−^ is generated in the presence of NO^−^ and Fe^3+^, the rate of carnosine-bound DNIC formation exceeds the rate of their destruction. The interaction of O_2_^•−^ with DNICs is thought to result in the formation of iron-bound peroxynitrite, which is further isomerized to nitrate [48,49], and these unstable intermediates can be converted back into DNICs. On the contrary, under carbonyl stress, carnosine can participate in the formation of DNICs capable of scavenging superoxide radicals along with its antiglycation effect [16].

Oxoferryl ([porphyrin^•+^-Fe^4+^=O^2−^]^2+^) and perferryl ([porphyrin^•+^-Fe^4+^=O^2−^]^2+^) heme forms resulting from hemoprotein interactions with H_2_O_2_ or organic peroxides are some of the most powerful oxidizing agents in biological systems [5,44,50,51,52]. These hypervalent Mb (myoglobin—the muscle hemoglobin) states are generated in the following reactions [50,52]:

Mb-Fe^2+^ + H_2_O_2_ → [Mb-Fe^4+^=O^2−^]^2+^ + H_2_O (1)

Mb-Fe^3+^ + H_2_O_2_ → [Mb^•+^-Fe^4+^=O^2−^]^3+^ + H_2_O (2)

**Figure 4:**
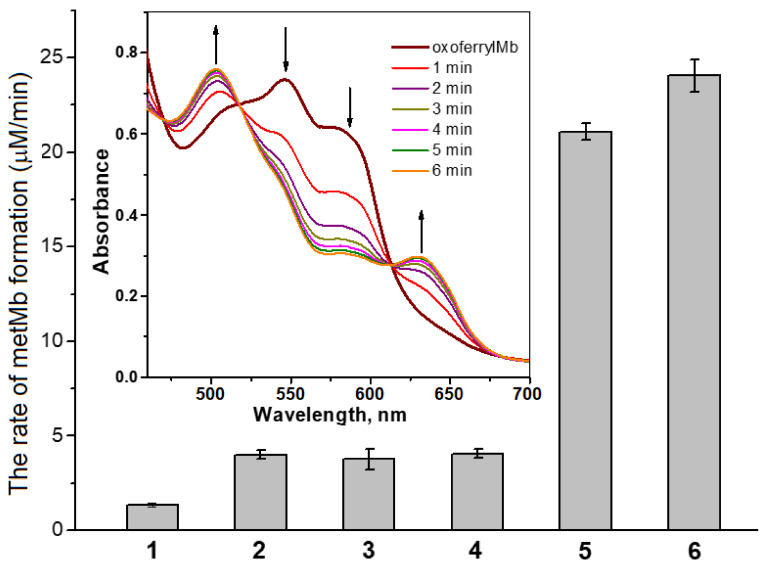
The rate of oxoferrylmyoglobin (oxoferrylMb) reduction to metmyoglobin (metMb) by DNICs with phosphate and carnosine ligands and by their decay products (NO_2_^−^ and Fe^2+^ ions). The reaction mixture: 0.1 M of HEPES (pH 7.5) and 92 μM of oxoferrylMb. 1—control (without additives). Additives: 2—0.5 mM of FeSO_4_; 3—1 mM of NaNO_2_; 4—1.25 mM of carnosine; 5—0.5 mM of carnosine-bound DNICs (obtained by blending 1.25 mM of carnosine with 0.5 mM of phosphate-bound DNICs); and 6—0.5 mM of phosphate-bound DNICs. Inset—absorption Mb spectra during the reduction of oxoferrylMb to metMb under the action of 0.5 mM of carnosine DNICs. The arrows indicate the decreases (556 and 583 nm) and increases (503 and 631 nm) in oxoferrylMb’s and metMb’s characteristic maxima.

The perferryl form is considered a combination of oxoferryl heme and the porphyrin cation radical. In our experiments simulating Reaction (1), H_2_O_2_ interacted with metMb to form oxoferrylMb. Glutathione-bound DNICs were earlier shown to effectively reduce oxoferrylMb to non-toxic metMb (Mb-Fe^3+^) [5]. Figure 4 shows the reduction of oxoferrylMb to metMb under the action of DNICs with carnosine and phosphate ligands. In these experiments, carnosine DNICs were obtained by adding phosphate DNICs to the carnosine.

The rate of oxoferrylMb reduction by DNICs containing phosphate and carnosine was considerably higher than that in the presence of nitrite and Fe^2+^ ions, which can be decay products of complexes (Figure 4). Carnosine itself also contributed significantly less to the neutralization of oxoferryl heme than DNICs containing this dipeptide. According to Herold and Rehmann, NO^•^ reduces Hb and Mb oxoferryl heme groups in the following reaction [53,54]:

[porphyrin-Fe^4+^=O^2−^]^2+^ + NO^•^ → porphyrin-Fe^3+^ + NO_2_^−^ (3)

The rate constant of this reaction is 1.8 × 10^7^ M^–1^s^–1^ [53]. NO^•^ is assumed to react with various free radicals resulting from the interaction of hemoproteins with hydroperoxides [5,44,49]. Therefore, the NO^•^ released from the complexes probably accounts for the observed DNICs’ antioxidant effect (Figure 4), while the direct interaction of the DNICs’ nitrosyl ligand with the oxoferryl heme cannot be excluded [5].

The interaction of hydroperoxides with hemoglobin (Hb) and Mb is known to generate free radicals from amino acid residues of these proteins in addition to oxoferryl heme, leading to their oxidative modification [6,49]. At the same time, oxoferryl heme groups and hemoprotein-associated free radicals can stimulate POL processes [44,51,52]. In the case of Mb, these processes occur in the muscle cells with the highest carnosine content [19,21].

### 2.2. Antioxidant and Antiradical Properties of DNICs Associated with Albumin 

**Figure 5:**
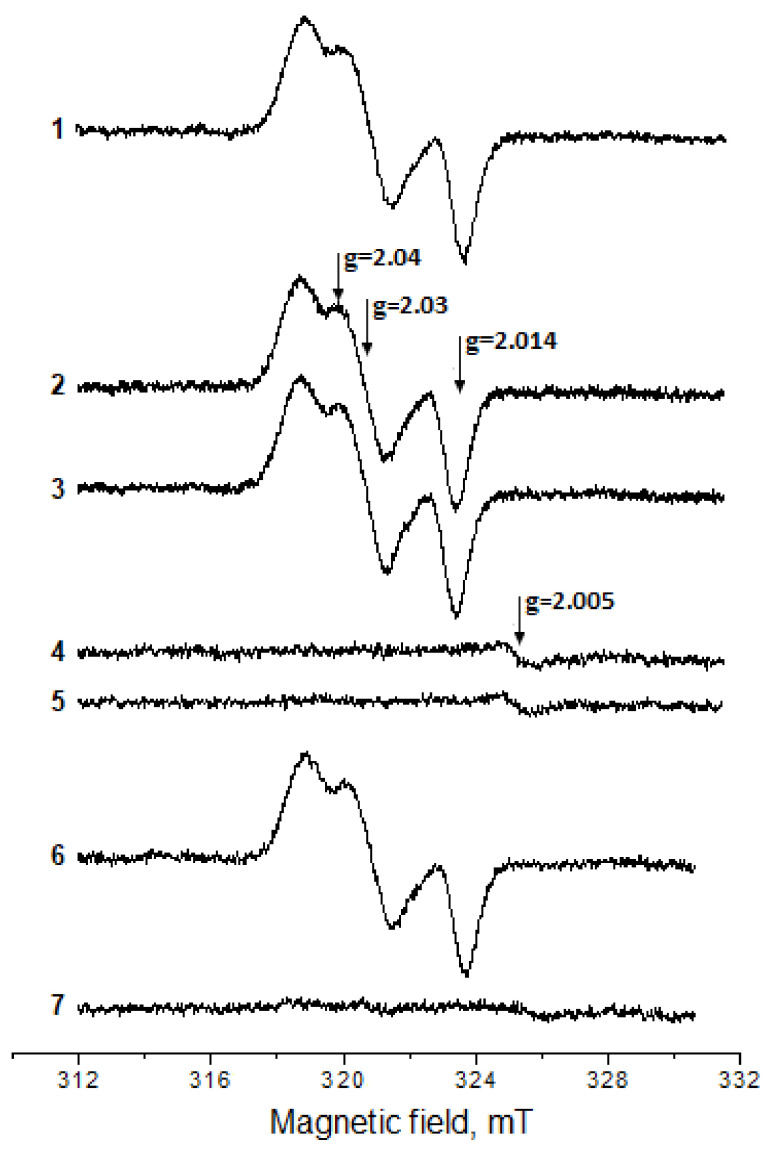
EPR spectra of the products of BSA-DNICs’ and metMb’s interactions with H_2_O_2_ or *t*-BOOH. The reaction mixture: 150 mM of Na,K-phosphate buffer (pH 7.4) and 360 μM of BSA-DNICs (1); same as (1) + 2.5 mM of H_2_O_2_ (2); same as (2) + 1 mM of DTPA (3); same as (3) + 100 μM of metMb (4); the reaction mixture contained only H_2_O_2_ and metMb (5); the same as (1) + 1 mM of DTPA + 2 mM of *t*-BOOH (6); and the same as (6) + 100 μM of metMb (7). The EPR spectra were recorded 4 min after all the components were mixed.

We have previously shown that DNICs associated with BSA (BSA-DNICs) are destroyed by superoxide radicals and peroxynitrite [6]. Herein, we studied the interaction of BSA-DNICs with *t*-BOOH and H_2_O_2_ in the presence and absence of metMb (Figure 5). BSA-DNICs themselves weakly reacted with hydroperoxides, which is consistent with the results obtained earlier [6].

At the same time, BSA-DNICs were completely destroyed via the interaction of hydroperoxides with metMb (Figure 5, spectra 4 and 7). In the reaction system containing metMb and H_2_O_2_, BSA-DNICs were most likely oxidized by the perferryl heme form produced in Reaction (2). The hydroxyl radical (^•^OH), formed in the Fenton reaction, catalyzed by contaminant iron can also participate in DNICs’ destruction. To prevent this reaction, the iron chelator DTPA was added to the mixture in most experiments, but it did not significantly affect the BSA-DNICs’ destruction (Figure 5, spectra 2–7). ^•^OH may be an intermediate of the reaction between the heme group and H_2_O_2_ [55,56,57], but the mechanism of hydroxyl radical formation in this reaction is still under discussion.

When metMb reacted with H_2_O_2_ in our experiments, the EPR signal of a stable free radical with a g-factor of 2.005 was registered (Figure 5, spectra 4 and 5). This radical was formed independently of BSA-DNICs’ presence and was probably a product of the one-electron oxidation of tyrosine and tryptophan residues in Mb [52,58,59].

**Figure 6:**
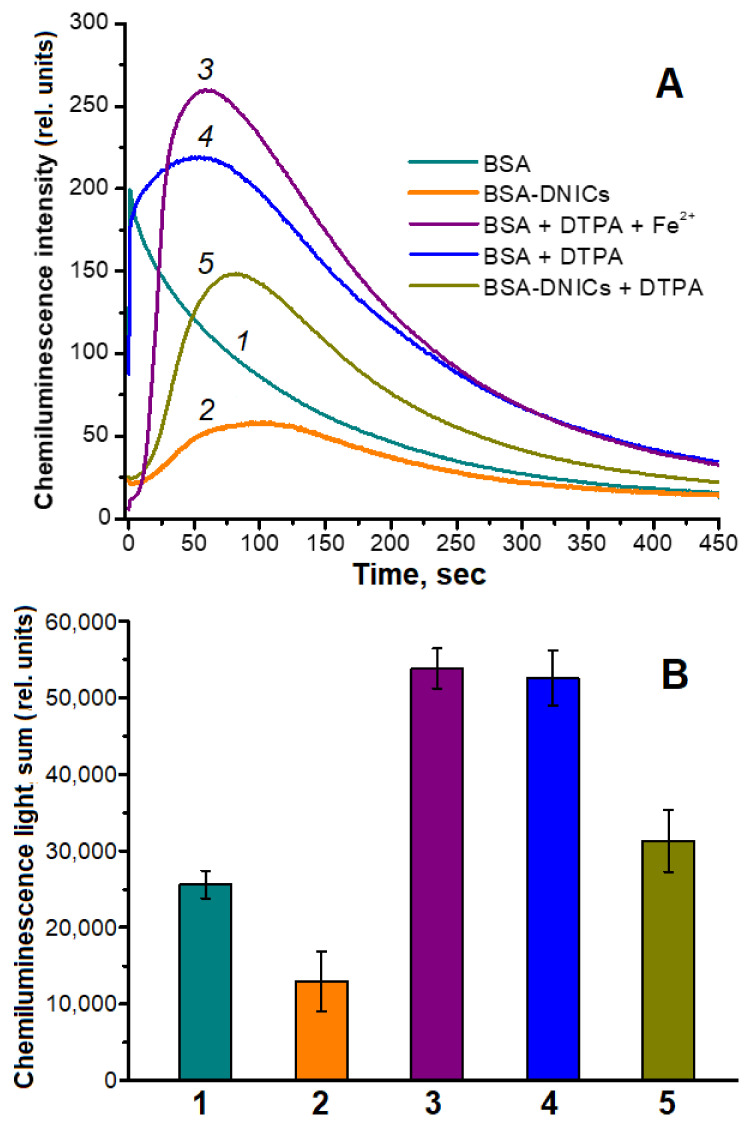
Effect of BSA-DNICs on luminol-dependent chemiluminescence induced by metMb oxidation with *t*-BOOH. (**A**)—kinetic curves of chemiluminescence in the reaction mixture containing 150 mM of Na,K-phosphate buffer (pH 7.4), 2 mM of luminol, 360 μM of bovine serum albumin (BSA), 100 μM of metMb, 200 μM of *t*-BOOH (1); the same as (1) but BSA is bound with DNICs (2); same as (1) + 1 mM of DTPA + 360 μM of FeSO_4_ (3); same as (1) + 1 mM of DTPA (4); and same as (2) + 1 mM of DTPA (5). (**B**)—chemiluminescence light sum (the area under the kinetic curve recorded in 450 s). Column labels correspond to the curves in panel (**A**).

The antioxidant effect of BSA-DNICs in a system modeling metMb peroxidation was studied using luminol-dependent chemiluminescence. In the BSA-DNICs-containing reaction mixture, the complexes significantly decreased the maximum intensity of chemiluminescence induced by metMb oxidation with *t*-BOOH (Figure 6A, curves 1 and 2). Likewise, in the presence of BSA-DNICs, the chemiluminescence light sum was two times lower than that in the sample with albumin (Figure 6B, columns 1 and 2). In addition, the kinetic chemiluminescence curve of the samples with BSA-DNICs had an induction period characteristic of many antioxidants [9,60]. These results indicate that BSA-DNICs effectively neutralized the pro-oxidants formed. Adding DTPA to the reaction mixture increased the chemiluminescence light sum (Figure 6B, columns 4 and 5). Analyzing the chemiluminescence kinetic curves allowed us to conclude that in the presence of the metal-ion chelator, the pro-oxidants were generated at a lower rate. Nevertheless, the antioxidant effect of BSA-DNICs in the system with DTPA remained (Figure 6B columns 4 and 5). Under these conditions, BSA-DNICs reduced the chemiluminescence light sum by 1.7 times. At the same time, the DTPA complexes with Fe^2+^ still participated in the generation of free radicals, interacting with *t*-BOOH (Figure 6A, curve 3), although the rate of this reaction was lower than in the cases of albumin-bound or free iron. Along with the dopant iron, the one released during the decay of Mb and DNICs hemes could have influenced the reaction in our experiments.

**Figure 7:**
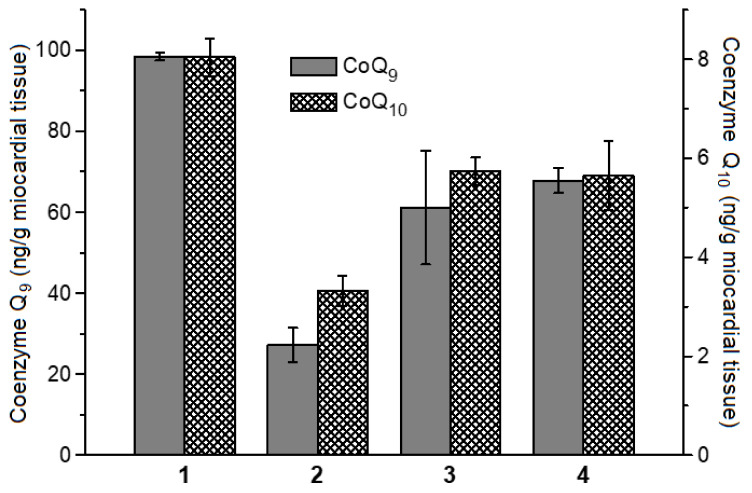
Changes in the levels of coenzymes Q_9_ and Q_10_ in rat myocardial homogenate after incubation for 90 min at room temperature without addition (1) or with addition of 1 mM of *t*-BOOH (2). Directly before adding *t*-BOOH, 600 µM of Angeli’s salt (3) or 140 µM of BSA-DNICs was added to the homogenate (4).

One of the most accurate methods for assessing the oxidative stress level is to determine the content of endogenous antioxidants in the biological sample. In the first stage of free radical oxidation in biological structures, which is the most similar to physiological conditions, the concentration of endogenous lipophilic antioxidants decreases. Along with its role in the bioenergetic processes in the cell, coenzyme Q (ubiquinone) is an important antioxidant protector in animals and humans [61,62]. Rats have two variants of this lipophilic antioxidant (coenzymes Q_9_ and Q_10_), which differ in the length of the isoprenoid tail [62]. We studied the effect of BSA-DNICs and the nitroxyl anion on the level of coenzymes Q in rat myocardial homogenate under the conditions of *t*-BOOH-induced free radical oxidation of lipids and other biomolecules (Figure 7). After a 90 min incubation with *t*-BOOH, the contents of coenzymes Q_9_ and Q_10_ in the rat heart homogenate decreased by 72 and 60%, respectively. In the presence of 140 μM of BSA-DNICs, the decrease in the coenzyme Q content was ~30%. An amount of 600 μM of Angeli’s salt had a comparable protective effect. Figure 7 also shows that coenzyme Q_9_ predominated in rat myocardial cells, which is in good agreement with the literary data [62,63,64]. 

Apparently, free radical oxidation in the homogenate is induced by several reactions, and *t*-BOOH free radical derivatives and hypervalent Mb forms play a special role in it. Under these conditions, Mb originates from damaged cardiomyocytes. The protonated nitroxyl anion HNO inhibits lipid peroxidation by reducing peroxyl (LOO^•^) and alkoxyl (LO^•^) radicals [4,65]:

LOO^•^/LO^•^ + HNO → LOOH/LOH + NO^•^ (4)

NO^•^ can also act as a potent antioxidant, breaking the POL chain processes in the following reactions [66,67,68]:

LOO^•^ + NO^•^ → LOONO → [LO^•^ + NO_2_^•^] → LONO_2_ (5)

LO^•^ + NO^•^ → LONO (6)

BSA-DNICs are donors of the nitric oxide producing their antioxidant effect, including the protection of coenzymes Q during free radical oxidation (Figure 7). This effect is consistent with a high reaction rate between peroxyl radical and NO^•^ (k = 1–3 × 10^9^ M^−1^ s^−1^) [67,68]. It should be noted that Reaction (5) generates an organic analog of peroxynitrite, ONOO, which can decompose to form free radicals such as LO^•^ and NO_2_^•^. BSA-DNICs are most likely to also react directly with the free radicals and oxoferrylMb.

## 3. Discussion

Our study shows that in various systems modeling oxidative stress, DNICs associated with histidine residues exhibit antiradical and antioxidant properties. In particular, BSA-DNICs, like carnosine DNICs, react with free radicals arising from *t*-BOOH oxidation. Carnosine DNICs effectively reduce pro-oxidant hypervalent Mb forms to metMb. These reactions determine the antioxidant properties of BSA-DNICs during the peroxidation of myocardial homogenate. We have previously shown that BSA-DNICs’ interaction with O_2_^•−^, peroxynitrite [6], and hypochlorous acid [7] is followed by their destruction. Carnosine DNICs were shown to be destroyed by the superoxide anion radical, but we demonstrated the regeneration of these complexes in the presence of NO^−^/HNO (Figure 3B). The reaction rate of the nitrosyl ligands of the studied DNICs with free radicals was significantly lower than that of free NO^•^. We have earlier shown a similar effect for the interaction of BSA-DNICs with O_2_^•−^ [6].

Since DNICs with carnosine and phosphate ligands reduce the hypervalent Mb forms with similar efficiency, NO^•^ can participate in the reaction with the heme group in a free form or bound in a complex (nitrosyl ligand). NO^•^ released from DNICs is able to react with alkoxyl and peroxyl radicals, thereby breaking off lipid peroxidation (POL) chain reactions. Another molecular mechanism of DNICs’ antioxidant action may be as follows: as a result of their interaction with O_2_^•−^ or lipid radicals, DNICs are transformed into intermediates and further disintegrated without forming strong oxidants. These intermediates may be iron complexes containing bound peroxynitrite or alkyl derivatives of peroxynitrite (LOONO) [9]. This complex containing ONOO was obtained via the interaction of molecular oxygen with DNICs liganded by synthetic nitrogen-containing compounds [69]. In addition, the binding of iron ions in DNICs inhibits the generation of free radicals in the Fenton and Haber–Weiss reactions, as well as the branching of POL reactions. These reactions are the main cause of cell death during ferroptosis [61,69,70,71]. In diabetic nephropathy, carnosine prevents the ferroptosis of renal tubule epithelial cells [72], which may be associated with DNIC formation. Antioxidant DNICs’ properties can be especially important in diabetes since hyperglycemic conditions result in oxidative stress, and, at the same time, the metabolic and signaling functions of NO^•^ are disrupted [16,49].

A number of diseases associated with oxidative stress and the necrotic death of myocardial cells manifest themselves in extracellular Mb and ischemically modified albumin [34,37,38,73,74]. The interaction of serum albumin and hypervalent Mb forms can occur during rhabdomyolysis and ischemic myocardial damage [74,75,76]. This process can also be associated with skeletal muscle ischemia. In some cases, under skeletal muscle ischemic damage, the level of oxidized ischemically modified albumin increases [74,77]. Meanwhile, during rhabdomyolysis, capillaries are damaged, and albumin is released into the extravascular space [76]. Taking all these facts into consideration, we investigated the antioxidant properties of BSA-DNICs under conditions mimicking metMb oxidation in solution and in rat myocardial homogenate.

In the reactions of hemoproteins with *t*-BOOH, the following organic radicals are formed together with the oxoferryl heme form [6,43,49,58,78]:

porphyrin-Fe^2+^-O_2_ + *t*-BOOH → porphyrin-Fe^3+^ + OH^−^ + *t*-BO^•^ + O_2_ (7)

porphyrin-Fe^3+^ + *t*-BOOH → [porphyrin^•+^-Fe^4+^=O^2−^]^3+^ + *t*-BOH (8)

porphyrin-Fe^3+^ + *t*-BOOH → [porphyrin-Fe^4+^=O^2−^]^2+^ + *t*-BO^•^ + H^+^ (9)

[porphyrin^•+^-Fe^4+^=O^2−^]^3+^ + *t*-BOOH → [porphyrin-Fe^4+^=O^2−^]^2+^ + *t*-BOO^•^ + H^+^ (10)

[porphyrin-Fe^4+^=O^2−^]^2+^ + *t*-BOOH → porphyrin-Fe^3+^ + *t*-BOO^•^ + OH^−^ (11)

Thus, the organic radicals, *t*-BOOH derivatives, can participate in the destruction of BSA-DNICs. In living organisms, alkoxyl and peroxyl radicals are formed in similar reactions during the interaction of heme groups with lipid hydroperoxides [43,59,78]. Due to the porphyrin cation radical in Reaction (10), the amounts of the protein-associated free radicals in the reaction system with *t*-BOOH and metMb were low. At the same time, in the system with hydrogen peroxide and metMb (Reaction (2)), the porphyrin cation radical oxidized the amino acid residues in Mb itself.

The nitroxyl anion (NO^−^) is a product of the single-electron reduction of NO^•^ in living systems, existing in equilibrium with its protonated form, nitroxyl (HNO) [4,79]. NO^−^/HNO is known to exhibit both pro-oxidant and antioxidant properties [1,4,80]. As a result of NO^−^ interacting with molecular oxygen in the following reaction, a strong oxidizer, peroxynitrite (ONOO^−^), is supposedly formed [1]:

NO^−^ + O_2_ → ONOO^−^ (12)

Furthermore, peroxynitrite occurs in a more widespread reaction between NO^•^ and the superoxide anion radical (O_2_^•−^) [2]:

NO^•^ + O_2_^•−^ → ONOO^−^ (13)

Then, peroxynitrite decays, giving rise to the hydroxyl radical (^•^OH) and nitrogen dioxide (NO_2_^•^), which cause the oxidative modification of various biomolecules.

On the contrary, NO^−^/HNO can intercept free organic radicals formed during lipid peroxidation (POL), inhibiting this process [4,80]. *t*-BOOH is often used in modeling oxidative stress and processes of free radical peroxidation of biomolecules [44,65,81,82]. When ferrous ions interact with *t*-BOOH, its homolysis proceeds according to the following reaction:

*t*-BOOH + Fe^2+^ → *t*-BO^•^ + OH^−^ + Fe^3+^ (14)

The products of Reaction (14) are *tert*-butoxyl radicals (*t*-BO^•^), analogs of lipid alkoxyl radicals. In biological systems, alkoxyl radicals are involved in the continuation of POL chains. Reaction (14) can also be considered as an analog of the Fenton reaction, in which Fe^2+^’s interaction with hydrogen peroxide forms ^•^OH.

Apparently, the binding of NO^−^/HNO when carnosine-bound DNICs are formed also regulates free radical oxidation processes. The increased production of active forms of NO^•^ is associated with numerous diseases [2,83]. In turn, carnosine protects cells from oxidative and nitrosative stress developing in a number of pathologies, including neurodegenerative diseases and inflammatory processes [25,27,30,83]. According to Nicoletti et al., the protective effect of carnosine on astroglial cells results from its ability to directly intercept NO^•^ and its active metabolites [83]. Meanwhile, the molecular mechanisms of NO^•^ and carnosine adduct formation remain unclear. It cannot be excluded that this mechanism involves reactions within complexes in which iron is bound to carnosine, nitrosyl, and ONOO^−^ ligands.

The data on carnosine’s effect on the formation and physiological activity of NO^•^ are contradictory. A number of studies showed that carnosine inhibited endothelial NO synthase (eNOS) and the expression of inducible NO synthase (iNOS) [30,84]. On the contrary, in other studies, carnosine stimulated the production of NO^•^ [30], in particular, by increasing the calcium level in endothelial cells, leading to eNOS activation [85]. Carnosine is also supposed to suppress the NO^•^-dependent activation of soluble guanylate cyclase by forming a complex with the heme iron of this enzyme [86]. We believe that the formation of carnosine DNICs allows us to explain both the ambiguous effects of carnosine on the metabolism coupled with the signaling function of NO^•^ and the cytoprotective properties of this dipeptide. For instance, DNICs can bind excessive NO^•^ produced by NO synthases as well as regulate NO^•^ interaction with the heme group of guanylate cyclase. Thus, DNICs can function as a kind of NO^•^ depot. DNICs associated with carnosine or histidine residues in proteins can better perform this function since the thiol ligands of DNICs are redox-active and can be oxidized [6,7]. According to Liu et al., glutathione DNICs release HNO under albumin action [87]. This fact indicates a possible relationship between thiol-containing DNICs, albumin, and nitroxyl, involved in the formation of carnosine DNICs.

DNICs associated with proteins may be responsible for the transport of NO^•^ in the blood’s circulatory system [12,13,49]. Meanwhile, during rhabdomyolysis, capillaries are damaged, and albumin is released into the extravascular space [76]. Thus, it can be assumed that DNICs associated with albumin act as antioxidants in blood plasma and tissue fluid, as well as in tissues under apoptosis and necrosis. Herewith, carnosine DNICs should primarily act as intracellular antioxidant protectors and regulators of free radical oxidation processes.

We cannot also exclude that in the homogenate of cardiac muscle, the nitroxyl anion turned into DNICs, whose ligands were carnosine. At the same time, the EPR signal of the carnosine DNICs after adding Angeli’s salt to the myocardial homogenate was not registered, which may be due to the relatively low concentration of carnosine in the heart muscle (no more than 0.1 mM) [19,21].

Thus, DNICs associated with carnosine and albumin play an important role in ROS and RNS metabolism. These complexes may be of particular importance for treating neurodegenerative diseases, diabetic hyperglycemia, and ischemic damage of muscle and other tissues.

## 4. Materials and Methods

### 4.1. Materials and Reagents

The following reagents were used: carnosine, bovine serum albumin (BSA), xanthine, xanthine oxidase (EC 1.17.3.2) from bovine milk, metmyoglobin from equine skeletal muscle (metMb), catalase (EC 1.11.1.6) from bovine liver, 4-(2-hydroxyethyl)piperazine-1-ethanesulfonic acid (HEPES), diethylenetriaminepentaacetic acid (DTPA), NaNO_2_, ferrous sulfate (FeSO_4_), ferric sulfate hydrate (Fe_2_(SO_4_)_3_·H_2_O), hydrogen peroxide (H_2_O_2_), *tert*-butyl hydroperoxide (*t*-BOOH), coenzyme Q_9_, coenzyme Q_10_, sodium borohydride (NaBH_4_), 5-amino-2,3-dihydrophthalazine-1,4-dione (3-aminophthalic hydrazide, luminol) (“Sigma-Aldrich” (St. Louis, MO, USA)); Angeli’s salt (sodium trioxidinitrate) (“Cayman Europe” (Tallinn, Estonia)); 4-hydroxy-(2,2,6,6-tetramethylpiperidin-1-yl)oxyl (4-hydroxy-TEMPO) (“Oxis” (Portland, OR, USA)).

### 4.2. Preparation of DNICs

Paramagnetic phosphate-bound DNICs were synthesized by treating 5.5 mM of FeSO_4_ in 100 mM of K-Na phosphate buffer (pH 6.8) with gaseous NO^•^ in a Thunberg tube containing 100 mL of the gas phase. An amount of 1 mL of FeSO_4_ solution in distilled water and 4.5 mL of phosphate buffer were placed in the bottom and upper parts of the Thunberg tube, respectively. The apparatus was vacuumed, and NO^•^ gas was added up to a 100 mm Hg pressure. The solutions were mixed and shaken (5 min), and then NO^•^ was evacuated from the Thunberg tube. DNICs bound with bovine serum albumin (BSA-DNICs) were prepared by adding phosphate-bound DNICs (to a final concentration of 0.4 mM) to the protein solution (0.55 mM) in 150 mM of K-Na phosphate buffer (pH 7.4). The obtained DNIC preparations were divided into portions, frozen, and stored in liquid nitrogen (77 K). 

DNICs associated with carnosine were synthesized before or during the experiment as described in [16]. These DNICs were formed in a reaction mixture containing the nitroxyl donor, Angeli’s salt, ferric ions (Fe^3+^), and carnosine, or by adding carnosine to phosphate DNICs.

### 4.3. EPR Measurements

EPR spectra were recorded with an E-109E spectrometer (“Varian”, Palo Alto, CA, USA) at room temperature (~25 °C) under the following conditions: microwave power—10 mW, microwave field frequency—9.15 GHz, and RF modulation amplitude—0.05–0.4 mT. The samples (80 μL) were placed in gas-permeable Teflon capillaries PTFE 22 (“Zeus Industrial Products”, Orangeburg, SC, USA) before measurements.

### 4.4. Production of Superoxide Anion Radical

The formation of the superoxide anion radical (O_2_^•−^) occurred as a result of one-electron oxygen reduction coupled with the oxidation of xanthine to uric acid catalyzed by xanthine oxidase (xanthine oxidoreductase, EC 1.17.3.2). O_2_^•−^ generation was initiated by adding xanthine oxidase (0.6 U/mL) to a reaction mixture containing 1.5 mM of xanthine and 0.1 M of HEPES buffer (pH 7.5).

### 4.5. Obtaining and Registration of Oxoferrylmyoglobin (OxoferrylMb)

Oxoferrylmyoglobin ([Mb-Fe^4+^=O^2−^]^2+^) was prepared by adding 1 mM of H_2_O_2_ to 0.1 mM of met-Mb in 100 mM of HEPES (pH 7.5). Catalase (EC 1.11.1.6) was added to the final dose of 400 units/mL after 3 min of incubation to remove the unreacted peroxide. The concentration of oxoferrylMb was calculated with the optical absorption at 421 nm (ε_421_ = 111 mM^–1^cm^–1^) [6,56]. Mb optical absorption spectra were recorded with a Cary 300 spectrophotometer (“VarianBio”, Naples, FL, USA) at room temperature in a 1 cm optical path length cuvette at a 600 nm/min scanning speed. Thereafter, DNICs with carnosine or phosphate ligands were added to the oxoferrylMb solution. The reduction of oxoferrylMb to metMb was studied based on the absorption spectra in the 450–700 nm range. The accumulation of met-Mb was monitored by the difference in absorption at 631 nm (one of the metMb maxima) and 680 nm (the nearest isobestic point). The rate of metMb formation was calculated as the tangent of the slope angle of the linear section on the kinetic curve of metMb formation.

### 4.6. Work with Laboratory Animals

The rats used in this study were of the “Wistar” line, bred in the vivarium of the E.I. Chazov National Medical Research Centre of Cardiology. The work with laboratory animals was carried out according to the International Recommendations on Biomedical Research of the Council for International Organizations of Medical Sciences (CIOMS) and the Order of the Ministry of Health and Social Development of the Russian Federation “On Approval of the Rules of Laboratory Practice” (no. 708n of 23 August 2019).

### 4.7. Preparation and Oxidation of Rat Heart Homogenate

The work with rats was carried out in the vivarium of the E.I. Chazov National Medical Research Centre of Cardiology. Male Wistar rats (weighing 250–300 g) were anesthetized via intraperitoneal injection of urethane (1.8 g of anesthetic per 1 kg of animal weight). Hearts were extracted, weighed, crushed, and mixed with 40 mM of Na,K-phosphate buffer (pH 7.4) in a ratio of 1:4. The mixture was homogenized in a glass homogenizer with Teflon pestle until a homogeneous mass was obtained. The protease inhibitor PMSF was added to the obtained myocardial homogenate, which was then diluted in a 1:10 ratio with a phosphate buffer. Its oxidation was initiated by adding 1 mM of *t*-BOOH. The homogenate aliquot (0.5 mL) was incubated for 90 min at room temperature (25 °C).

### 4.8. Determination of Coenzymes Q_9_ and Q_10_ Contents in Rat Myocardial Homogenate

The coenzyme Q content in the rat myocardial homogenate was quantitatively analyzed according to [88]. An amount of 220 μL of ethanol and 550 μL of *n*-hexane were added to the myocardial homogenate sample (100 μL). The mixture was thoroughly shaken for 10 min and centrifuged at 3000 rpm for 3 min, and 500 µL of the upper hexane phase was sampled. The remains were repeatedly extracted with 550 µL of *n*-hexane. The combined extract was evaporated and redissolved in 100 µL of ethanol, and then 10 µL of a 5% NaBH_4_ solution in ethanol was added to reduce the oxidized forms of the coenzymes Q. The coenzyme Q_9_ and Q_10_ concentrations were determined via HPLC using the equipment and software of “Environmental Sciences Associates” (San Francisco, CA, USA). The chromatography was performed in isocratic mode in a Luna 150 × 4.6 mm column with C-18 sorbent (5 μm). The mobile phase was 0.3% NaCl in a mixture of ethanol/methanol/7% HClO_4_ (970:20:10). Electrochemical detection was carried out using an analytical cell (model 5011) under oxidative conditions (a −50 mV voltage on the first pair of electrodes and +50 mV on the second).

### 4.9. Determination of DNICs’ Antioxidant Activity with Chemiluminescence

The formation of free radical intermediates in Mb’s reaction with *t*-BOOH was chemiluminescently evaluated using luminol as an activator. Chemiluminescence was registered using a Lum-100 chemiluminescence analyzer (DISoft, Moscow, Russia) starting from 5–7 s after mixing the components of the reaction mixture. The reaction medium for studying the antioxidant activity of BSA-DNIC contained 0.1 mM of metMb in 150 mM of K-phosphate buffer (pH 7.4) + 0.04 mM of BSA/BSA-DNIC + 2 mM of luminol + 0.20 mM of *t*-BOOH, while that for studying the antioxidant activity of carnosine-DNIC consisted of 0.1 mM of metMb in 150 mM of K,Na-phosphate buffer (pH 7.4) + 1.5 mM of carnosine/carnosine-DNIC in 0.03 mM of HEPES (pH 7.6) + 2 mM of luminol + 0.2 mM of *t*-BOOH. In some variants, 1 mM of DTPA (metal chelator) or 1 mM of DTPA with FeSO_4_ was added in a concentration equivalent to the Fe^2+^ content in DNICs.

### 4.10. Statistical Analysis

The measurements were performed in at least three replicates for each sample. The statistical data were processed based on 3–4 analytical repetitions. The data are presented as means ± standard deviation. The ANOVA statistical model and Student’s *t*-test were used for the obtained data analysis.

## 5. Conclusions

DNICs associated with natural compounds are a physiological form of deposition and transport of NO^•^. Glutathione DNICs are the most studied. DNICs with non-thiol biologically active ligands, such as imidazole ligands, may also be of interest to medicine. Replacing a thiol ligand with an imidazole ligand makes the complexes more redox-resistant. These histidine-bound DNICs are based on peptides and proteins with available imidazole groups, e.g., BSA [16] and carnosine [16].

Despite the numerous known beneficial effects of DNICs in biological systems, the mechanisms underlying the effect of these complexes on the free radical peroxidation of biomolecules are not fully understood. Our results indicate the antioxidant and antiradical action of histidine-bound DNICs. Possible molecular mechanisms of this action are being discussed. These results may be a prerequisite for implementing the studied DNICs in clinical practice for the treatment of diabetes, cardiovascular, and oncological diseases.

## Data Availability

The data presented in this study are available in this article.
